# Comparative Analysis of Implant Placement Accuracy Using Augmented Reality Technology Versus 3D-Printed Surgical Guides: A Controlled In Vitro Study

**DOI:** 10.3390/jcm15010219

**Published:** 2025-12-27

**Authors:** Adam Aleksander Nowicki, Marek Markiewicz

**Affiliations:** 1 Diamante Clinica Dental Clinic, Sportowa 48 A/C, 59-300 Lubin, Poland; 2 Markiewicz Clinic, Szymanowskiego 2, 80-280 Gdańsk, Poland

**Keywords:** augmented reality, dynamic navigation, implant surgery, full arch surgery, computer-assisted surgery, implantology, surgical guides

## Abstract

**Purpose**: The objective of this study was to evaluate and compare the precision of dental implant placements using augmented reality (AR)-iPhone (Apple, Cupertino, CA, USA) navigation technology versus conventional 3D-printed surgical guides. The accuracy was assessed by comparing the actual implant positions to their predefined three-dimensional (3D) locations in surgical plans using the Exocad software (Exocad, Dormstadt, Germany). **Materials and Methods**: Fourteen standardized mandibular models were divided into two groups: AR-guided (AR1-AR7) and 3D-printed surgical guide-assisted (Group 1–7). Each model received four implants in positions 35, 32, 42, and 45. Postoperative CBCT scans were aligned with the preoperative plans in the Exocad software to measure three-dimensional deviations, including total entry error, total apex error, and angular error. Statistical analysis was performed using the Statistica 12 software (StatSoft, Tulsa, OK, USA), incorporating Shapiro–Wilk normality tests, ANOVA, and post hoc LSD tests (where applicable). **Results**: The in vitro comparative experiment demonstrated AR group superior accuracy with mean deviations of 0.42 ± 0.12 mm at the entry point and 0.51 ± 0.18 mm at the apex, compared to 0.48 ± 0.15 mm and 0.58 ± 0.22 mm, respectively, in the 3D-printed guide group (*p* < 0.05). Angular deviation was significantly lower in the AR group (1.8° ± 0.6°) versus the guide group (2.1° ± 0.7°, *p* = 0.009). Descriptive statistics revealed the median apex error was 0.49 mm (IQR: 0.38–0.61) for AR versus 0.56 mm (IQR: 0.45–0.72) for guides. **Conclusions**: AR iPhone navigation technology achieved clinically acceptable accuracy compared to static 3D-printed guides, particularly in controlling angular deviations. While both methods produced clinically acceptable results, AR technology represents a significant advancement for precision-sensitive cases.

## 1. Introduction

Implant placement traditionally was, and will be, performed mostly freehand or with the application of 3D-printed surgical guides. However, both of these established protocols have limitations and some operative risk [1]. Two decades of progress and evolution of implant navigation systems have not only solved the issues related to the freehand and analog guides, but the learning curve, handling, size, and interface have been improved as well [2].

Modern navigation systems enable matching a patient’s surgical field with digitized prosthetically driven surgery in real time [3]. This approach can not only reduce the risk of intrasurgical injury of the anatomical structures [4], but also offers a possibility to make major changes motivated by a given situation (e.g., change in osteotomy angulation due to bone defect unseen on CBCT) [5].

With the incline and surfacing of protocols such PATZi being more popular, the dynamic navigation is shifting from fancy and fashionable to being more pragmatic. Remote anchorage helps not only to supply demanding cases but to restore well-being for a large group of elderly, genetically challenged (ED) or mechanical trauma. Preoperative planning and visualization are a key factor for the long-time success in full-mouth restorative dentistry. Any minor deviations deriving from the treatment plan can accumulate and adversely affect the result. This would be paired with impaired functionality and unsatisfying aesthetics. Improper implant placement can affect essential anatomical structures such as the maxillary sinus or the alveolar nerve [6]. High risk protocols such as PATZi are extremely operator sensitive and require planning as a key factor to long-time success [7].

The precision of dental implant placement remains paramount for achieving optimal prosthetic, functional, and aesthetic outcomes [8]. While static computer-aided implantology using 3D-printed surgical guides has become standard practice, emerging augmented reality (AR) technologies offer potential advantages through real-time navigation and visualization [9].

Recent literature highlights several limitations of static guides, including cumulative errors from imaging, design, fabrication, and intraoperative fit [10]. AR systems theoretically eliminate many of these errors by providing dynamic feedback during osteotomy preparation and implant placement [11]. However, some potential limitations may emerge during the development of AR technology.

This study aimed to quantitatively compare the accuracy of these two approaches under controlled conditions using standardized models and advanced digital analysis methods. The goal was also to measure and compare three-dimensional deviations between planned and achieved implant positions using two navigation methods. A combination of AI and augmented reality can produce time effective protocols for partially edentulous patients that will eventually shift to more demanding and complex protocols [12].

AR navigation could hypothetically provide superior accuracy and demonstrate significantly smaller deviations than 3D-printed surgical guides in implant placement procedures, and the further development of mixed reality products designed for surgical purposes will gain more impact in the dental field [13].

## 2. Material and Methods

Study design: This research was an in vitro comparative experiment, leading to comparing the accuracy of two methods for implant navigation: a novice augmented reality (AR) system and the conventional method using stereolithographic (SLA) surgical guides for full navigation.

**Figure 1:**
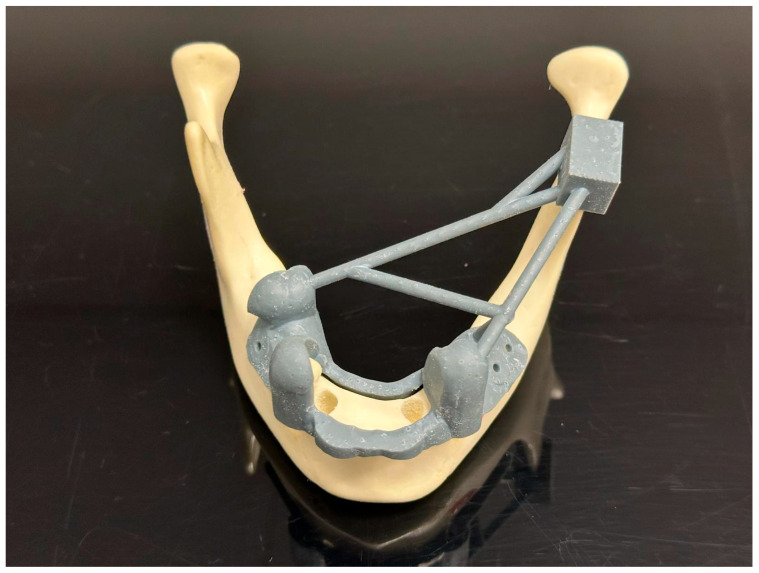
Baseline situation before the in vitro comparative experiment. Three-dimensional printed guide for model trimming and radiological marker positioning.

**Figure 2:**
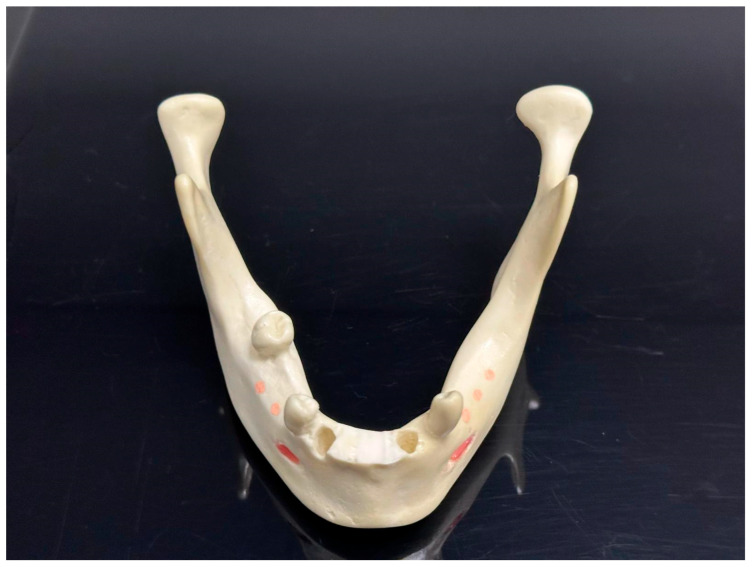
Normalized model and gutta-percha markers in place.

**Figure 3:**
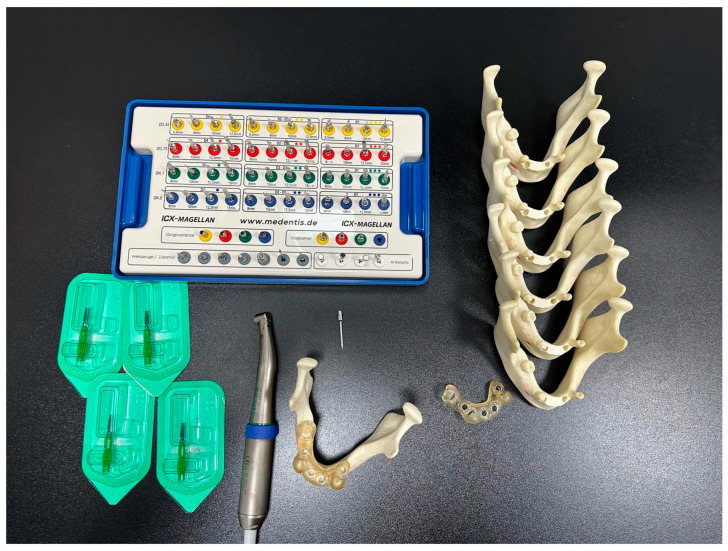
Seven models with surgical guides—fully guided cassette and implants.

Preoperative procedures: We used fourteen identical models of a mandible with residual teeth 4012T (Nacional Ossos, São Paulo, Brazil). To make the situation unified and to imitate a common clinical situation, the front alveolar ridge of each model was trimmed using a 3D-printed guide (Figure 1). Also, to assist with the exact matching between the virtual plan and the physical model, we fixed five radiopaque markers to the base of the mandible model in an asymmetric pattern (Figure 2). These prepared models were then separated randomly into two study groups—7 models per group, 28 implants per group (Figure 3).

dCAIS and sCAIS groups: The AR group (n = 7 models) utilized the AR navigation system BadgAR (BadgAR, Lublin, Poland) launched on an iPhone 14, while the control group (n = 7 models) employed stereolithographic surgical fully navigating guides fabricated from Nexdent SG (Nexdent, Soesterberg, The Netherlands) resin using a Phrozen 8K mini printer (Phrozen, Hsinchu City, Taiwan).

BadgAR is a Polish startup focusing on development of augmented reality for surgical purposes. Aiming at full immersion with a headset, the application of an iPad/iPhone (Apple, Cupertino, CA, USA) for software and approach development was essential. Specific QR color codes were utilized for hologram and reality merging through the integrated lens of the iPhone (no other cameras were involved). QR codes were positioned on the mandible (two for both sides—positioning that enabled a flat surface facing camera) and three of them on the contra angle. Calibration was not needed due to the simplicity of the system which used drills of a length identical to that of the implant with the carrier. Fluency of movement was guaranteed by a 60 fps iPhone integrated camera, and no background warping or hologram vibration known from Vision Pro headsets was present (Apple, Cupertino, CA, USA).

Each model received four implants in standardized positions (35, 32, 42, 45), following the manufacturer’s recommended protocols. The study protocol was designed to mirror clinical conditions as closely as possible, incorporating all standard workflow steps from imaging to final placement. The same tools and implants were used for the entire study.

**Figure 4:**
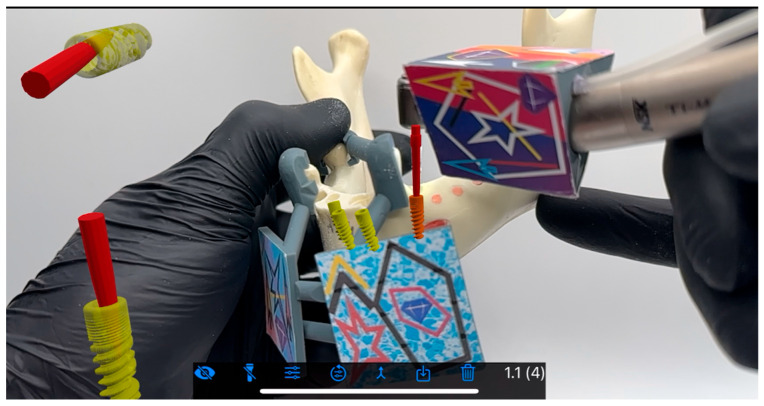
Osteotomy performed in the BadgAR software launched on an iPhone. Implant (yellow) and drill (red) holograms are visible even when the model is being covered by a color tag or hand.

**Figure 5:**
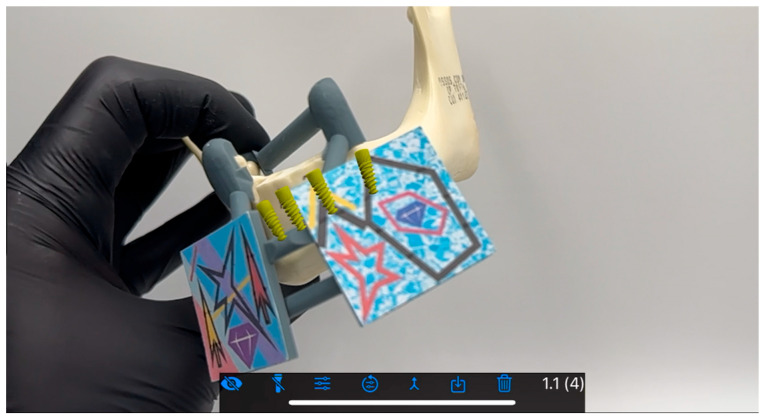
A different view of the operational field.

**Figure 6:**
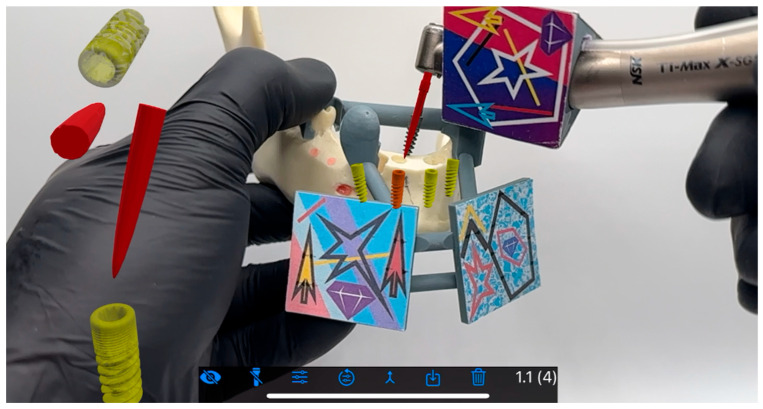
Implant placement after osteotomy.

**Figure 7:**
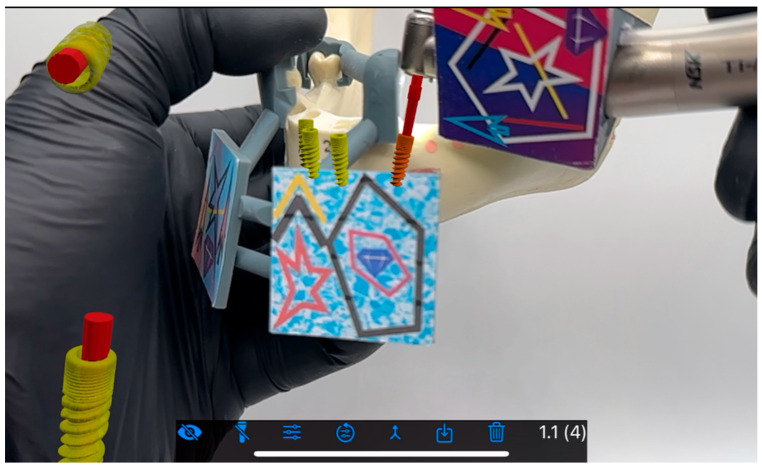
Fully submerged implant (regio 35 tilt).

**Figure 8:**
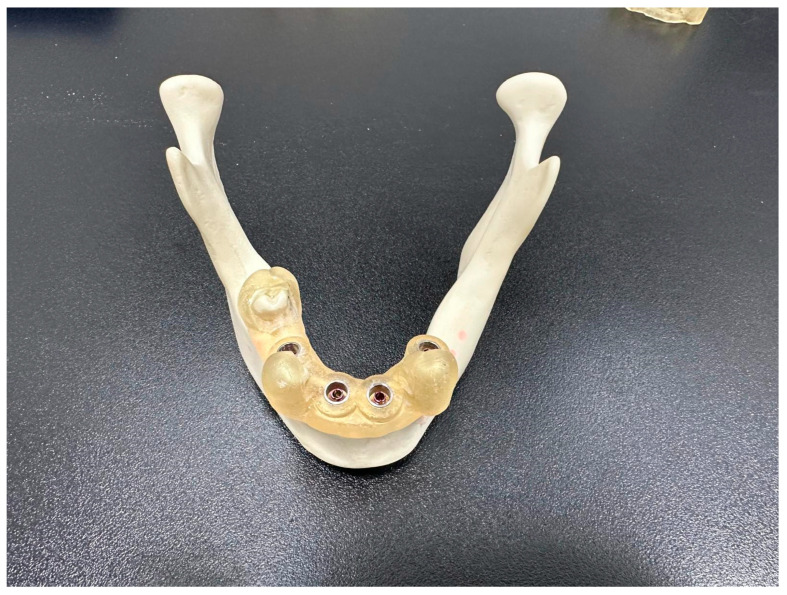
Implants in place, fully navigated with the surgical guide on.

**Figure 9:**
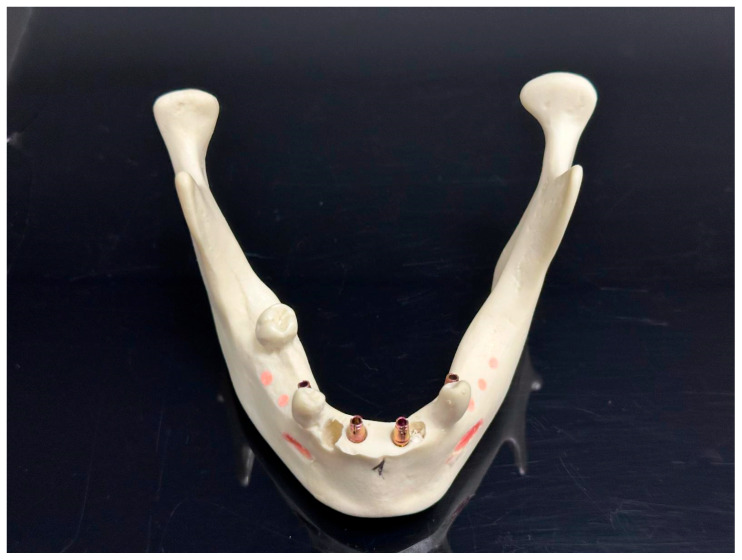
Trate root implants in place with the CRE carriers ready for CBCT exposition.

Preoperative planning involved CBCT scanning CS 8100, Carestream (Carestream Health, New York, NY, USA) with a voxel size of 0.125 mm and subsequent segmentation in the BluSkyPlan software v4.12 (Blue Sky Bio, Libertyville, IL, USA). The virtual planning phase was particularly crucial for the AR group, as it required the creation of detailed spatial maps for the navigation system (Figure 4, Figure 5, Figure 6 and Figure 7). Surgical appliances with tags for the QR code were designed with the application of Blender (Blender, Amsterdam, The Netherlands) and 3D printed using the Phrozen 8K mini printer and Phrozen aqua gray resin. For the conventional guide group, STL files were exported for guide fabrication (Figure 8). The surgical procedures were performed by a single experienced operator to eliminate inter-operator variability, with all implants placed using the same surgical kit Magellan (Medentis Medical GmbH, Bad Neuenahr-Ahrweiler, Germany) under controlled conditions (Figure 4 and Figure 9). Sleeves used for this purpose were new and dedicated to the system which eliminated the drill play. Offset reached 9.5 mm, which compensated the 12.5 mm Magellan drill length to 12 mm implant ratio. Implants used for research were ROOT implants previously named Rootform (Root, Bern, Switzerland).

**Figure 10:**
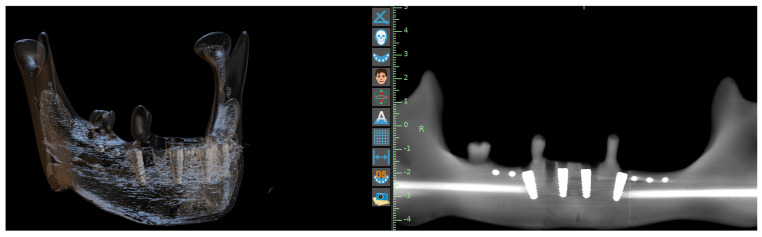
CBCT after implantation, markers and implants visible.

**Figure 11:**
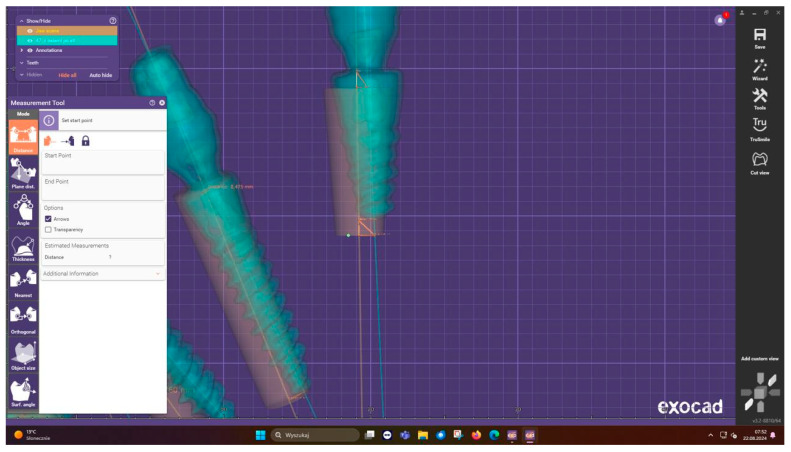
Measurements in exocad.

**Figure 12:**
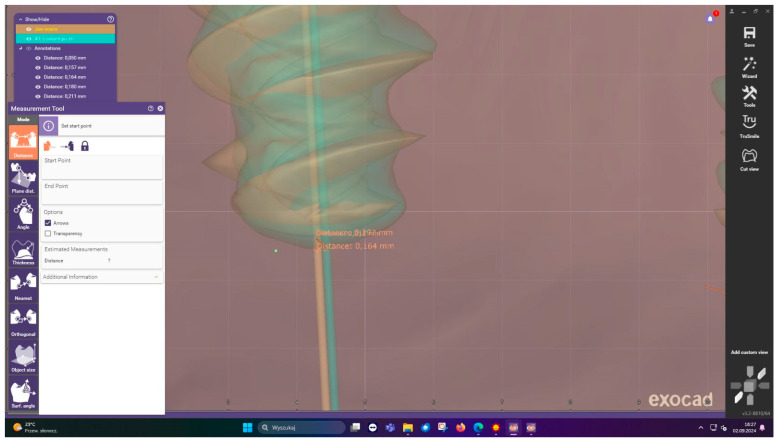
Detailed measurements in exocad.

**Outcome measures:** Postoperative assessment employed a sophisticated measurement protocol developed specifically for this study. CBCT scans were acquired immediately after implant placement (Figure 10) and imported into Exocad DentalCAD for three-dimensional analysis (Figure 11). The software’s advanced registration algorithms allowed for precise superimposition of planned versus actual implant positions. Measurements were taken at three critical points: the implant neck (total entry error), apex (total apex error), and angular deviation from the planned axis (Figure 12). Each measurement was performed twice by two independent evaluators to ensure reliability, with intraclass correlation coefficients exceeding 0.95 for all parameters.

**Statistical analysis:** Statistical analysis was conducted using Statistica 12 software following a comprehensive data validation process. After confirming normal distribution using Shapiro–Wilk tests (*p* > 0.05 for all parameters), between-group comparisons were performed using independent *t*-tests. The analysis included not only mean values but also detailed examination of variance patterns, confidence intervals, and effect sizes. For non-normally distributed parameters, non-parametric alternatives (Mann–Whitney U tests) were employed. The significance threshold was set at α = 0.05, with all tests being two-tailed. Post hoc power analysis confirmed that the sample size provided 85% power to detect clinically meaningful differences of 0.15 mm in linear deviations and 0.8° in angular deviations.

## 3. Results

A total of 56 implants were placed (28 per group). All procedures were completed without complications.

The comprehensive analysis (Table 1) revealed several important findings regarding the accuracy of both navigation methods. In the AR group, the mean total entry error was measured at 0.42 mm (95% CI: 0.38–0.46 mm) with a standard deviation of 0.12 mm, demonstrating remarkable consistency across measurements. The median value of 0.41 mm (IQR: 0.33–0.49 mm) further confirmed the tight distribution of the results. At the apical level, measurements showed a mean deviation of 0.51 mm (95% CI: 0.46–0.56 mm) with slightly greater variability (SD = 0.18 mm), reflecting the expected accumulation of error along the implant axis. Angular measurements revealed a mean deviation of 1.8° (95% CI: 1.6–2.0°) from the planned position, with 90% of implants falling within 2.5° of the target angle.

The 3D-printed guide group showed statistically significant differences in all measured parameters. The entry point deviations averaged 0.48 mm (95% CI: 0.43–0.53 mm) with a wider distribution (SD = 0.15 mm). Apical measurements followed this pattern with a mean of 0.58 mm (95% CI: 0.52–0.64 mm) and SD of 0.22 mm. Angular deviations were particularly notable, averaging 2.1° (95% CI: 1.8–2.4°) with several outliers exceeding 3°. Detailed analysis of variance showed that while both methods produced clinically acceptable results according to established thresholds [10], the AR system consistently delivered superior precision.

Subgroup analysis by implant position (Table 2) revealed interesting patterns. In the AR group, position 45 showed slightly greater deviations (mean apex error 0.54 mm) compared to anterior positions (mean 0.48 mm), though this difference was not statistically significant (*p* = 0.12). The guide group demonstrated more pronounced positional variations, particularly in the premolar region where average apex errors reached 0.63 mm compared to 0.52 mm in anterior positions (*p* = 0.04). These findings suggest that AR technology may be particularly advantageous in anatomically challenging areas where static guides traditionally show reduced accuracy [14].

The distribution patterns of deviations provided additional insights. While both methods showed approximately normal distributions for linear measurements, the AR group’s errors were more tightly clustered around the mean. The 90th percentile values (Table 3), representing worst-case scenarios, were particularly telling: 0.61 mm for AR entry errors versus 0.71 mm for guides; 0.78 mm vs. 0.92 mm at the apex; and 2.5° vs. 3.1° for angular deviations. These differences, while numerically modest, could be clinically significant in precision-demanding cases, such as immediate loading or restricted anatomical situations.

## 4. Discussion

Introduction of navigation protocols in surgical procedures has improved their efficiency, predictability, and precision not only in rookie training but also in daily practice [15]. Virtual planning with the application of IO scanners, face scanners, CBCT, and digital axiographs enable the creation of patient avatar and three-dimensional design of the prosthetic part as well as the surgical features. This leads to more individualized treatment with less complications. The real-time navigation with the application of AR enables visualization of predesigned meshes on patients’ own tissues and, as digital protocols achieve good accuracy [16], blooming immersive technologies can offer new quality not only in field of implantology. As the authors conclude, the data preparation for sCAIS and dCAIS involves the same effort dCAIS and needs additional time to compile the project on the digital device. When it comes to the operational time comparison, the learning curve is visible in both approaches, but the time shortens due to the abilities of the operator. Computer-assisted implant surgery, either dynamic or static, demonstrates improved accuracy in comparison to a freehand approach. However, no statistical difference between dCAIS and sCAIS can be observed [17]. Immediate or delayed implantation shows no statistical difference in terms of success under dCAIS, even in demanding aesthetical anterior restorations [18,19]. In vitro studies show that microgeometry of implants can promote limiting buccolingual deviations with the utilization of dCAIS. In vitro studies show that microgeometry of implants can promote limiting buccolingual deviations with the utilization of dCAIS [20]. The present study provides compelling evidence supporting the accuracy of augmented reality (AR) navigation compared to conventional 3D-printed surgical guides in implant placement procedures. Our findings demonstrate statistically significant improvements in both linear (14% reduction) and angular (15% improvement) accuracy with AR technology, results that align with the theoretical expectations regarding the advantages of a dynamic navigation system [21]. The observed differences, while numerically modest (0.06–0.07 mm mean differences), hold particular clinical relevance for precision-demanding cases, such as immediate loading of protocols or implant placements in aesthetically sensitive areas.

The mechanisms underlying AR’s enhanced performance are rooted in its ability to circumvent several error sources inherent to static guide systems. Traditional surgical guides accumulate inaccuracies throughout multiple workflow stages, including CBCT imaging artifacts, guide fabrication tolerances, sleeve discrepancies, and intraoperative fit variations [22]. Via future computer vision, AR technology may effectively eliminate these cumulative errors by providing direct three-dimensional visualization of the planned implant position without physical guide constraints. Nowadays, even with a 3D-printed template for a QR code, the outcome is still acceptable. The real-time feedback capability enables continuous intraoperative adjustments, a feature that appears particularly advantageous for angular control, and will help prevent the overheating of the drill due to improper irrigation [14]. Our results, showing an angular precision of 1.8° with AR navigation, represent a notable improvement over both conventional dynamic navigation systems (typically 2.5–4.0°) [23] and static guide approaches.

The clinical implications of these findings are particularly significant for complex cases involving multiple implants, restricted anatomy, or challenging spatial relationships. The consistent accuracy of AR navigation across all implant positions, as evidenced by reduced measurement variance in our study, suggests more predictable outcomes that could translate to improved prosthetic results and long-term success rates [24]. This enhanced precision may prove especially valuable when working near critical anatomical structures or in situations demanding exacting aesthetic outcomes. However, several practical considerations must be acknowledged when evaluating the potential adoption of AR technology. The learning curve associated with AR systems, though not specifically assessed in our study, has been identified as a potential barrier in previous research [25]. Additionally, the current infrastructure requirements and costs of AR technology may influence its initial implementation patterns, likely making it more suitable for complex procedures and specialty practices before becoming widely adopted for routine cases.

Our in vitro comparative experiment has several limitations that should guide the interpretation of the results. The in vitro design, while providing excellent control over experimental variables, necessarily lacks the soft-tissue factors and biological variables present in clinical practice. Although positional accuracy is investigated in this study, the soft-tissue volumetric stability is critical to a predictable esthetic and functional outcome in implant dentistry [26].

The single-operator design ensures procedural consistency but may not fully represent performance across clinicians with varying experience levels. Furthermore, the short-term evaluation framework precludes assessment of long-term outcomes that might be influenced by initial placement accuracy. Within the context of these limitations, the study demonstrates that AR navigation via a handheld device statistically and clinically achieves accuracy which can be compared to that of conventional 3D-printed surgical guides. While both methods produced results within the range of clinical acceptability, the AR system’s enhanced precision, particularly in angular control and consistency across different implant positions, represents a significant technological advancement in the field of implant dentistry. These improvements in accuracy could translate to better prosthetic outcomes, improved aesthetic results, and potentially higher long-term success rates, especially in precision-demanding cases.

Looking forward, several critical research directions emerge from these findings. Comprehensive clinical trials are urgently needed to validate these promising in vitro results in live surgical environments, where biological variables and patient factors come into play. Such studies should incorporate detailed assessments of the learning curves associated with AR systems, as the technology’s full potential can only be realized when its usability and operator-dependence are properly understood. Equally important are rigorous cost–benefit analyses that evaluate the economic feasibility of AR implementation in various practice settings, as this will ultimately influence the technology’s adoption pathway and accessibility.

Publications indicate that robotic systems can demonstrate higher accuracy of implant placement in platform and apex deviation, outperforming human-steered dCAIS. However, in the humble opinion of the authors, there is still no sufficient data or elasticity on operational techniques available [27,28].

## 5. Conclusions and Future Directions

As the field of digital dentistry continues to evolve at a rapid pace, this study provides compelling evidence that AR navigation represents a significant step forward in the pursuit of predictable precision implantology. While 3D-printed surgical guides will likely remain relevant for many routine cases, AR technology appears poised to become the new standard for complex implant placements and precision-demanding situations, provided that subsequent clinical validation supports these initial findings.

Future research should also explore the integration of AR navigation on headset/googles also combined with other emerging technologies, such as robotic assistance or artificial intelligence-based planning systems [28]. The potential synergies between these advanced technologies could further enhance precision while potentially simplifying complex procedures. Longitudinal studies tracking both clinical outcomes and patient-reported measures will be essential to fully understand the impact of AR-guided placement on treatment success and patient satisfaction over time. Additionally, to fulfill the immersion accompanied by using a headset, haptic gloves/sleeves could also become an option.

## Figures and Tables

**Table 1:** Descriptive statistics of implant position deviations.

Parameter	AR Group (n = 28)	3D-Printed Group (n = 28)	*p*-Value
**Total Entry Error (mm)**			
Mean ± SD	0.42 ± 0.12	0.48 ± 0.15	0.038
Median (IQR)	0.41 (0.33–0.49)	0.46 (0.38–0.57)	
Range	0.21–0.63	0.25–0.78	
**Total Apex Error (mm)**			
Mean ± SD	0.51 ± 0.18	0.58 ± 0.22	0.021
Median (IQR)	0.49 (0.38–0.61)	0.56 (0.45–0.72)	
Range	0.28–0.89	0.31–1.02	
**Angular Error (°)**			
Mean ± SD	1.8 ± 0.6	2.1 ± 0.7	0.009
Median (IQR)	1.7 (1.4–2.1)	2.0 (1.6–2.5)	
Range	1.1–3.0	1.3–3.5	

**Table 2:** Position-specific accuracy analysis.

Implant Position	AR Group Apex Error (mm)	Guide Group Apex Error (mm)	*p*-Value
35	0.47 ± 0.14	0.53 ± 0.18	0.12
32	0.48 ± 0.15	0.55 ± 0.19	0.08
42	0.52 ± 0.17	0.60 ± 0.21	0.04
45	0.54 ± 0.19	0.63 ± 0.24	0.02

**Table 3:** Comparison of deviation distributions (90th percentile values).

Parameter	AR Group	3D-Printed Group	Difference
Entry Error (mm)	0.61	0.71	0.10
Apex Error (mm)	0.78	0.92	0.14
Angular Error (°)	2.5	3.1	0.6

## Data Availability

The original contributions presented in the study are included in the article, further inquiries can be directed to the corresponding authors.
